# Does Attending Mainstream School Improve the Social Inclusion of Children on the Autism Spectrum and their Parents? A Cross-Sectional Study in China

**DOI:** 10.1007/s10803-025-06774-3

**Published:** 2025-03-01

**Authors:** Binbin Ji, Xinyi Peng, Lu Hong, Yoko Shimpuku, Chie Teramoto, Sanmei Chen

**Affiliations:** 1 School of Nursing, Hunan University of Chinese Medicine, Hunan Province 410208 Changsha, China; 2 Division of Nursing, Graduate School of Biomedical and Health Sciences, Hiroshima University, 1-2-3 Kasumi, Minami Ward, Hiroshima, 734-8553 Japan

**Keywords:** Autism, Children, Parents, Social inclusion, Mainstream school

## Abstract

This study investigated whether attending mainstream school is associated with improved social inclusion among children on the autism spectrum and their parents. The cross-sectional study took place from July to August 2023 at 30 rehabilitation centers for children on the autism spectrum in Hunan Province, China. Participants included 860 children aged 3–14 years, with autism history receiving outpatient rehabilitation services, and their primary caregiver parents aged 23–54 years. Social inclusion among children on the autism spectrum was evaluated using the Chinese version of the social inclusion subscale of the KidsLife-ASD Scale. Parental social inclusion was assessed using the Chinese version of the Social Inclusion Scale. Linear mixed models were used to explore the association between attending mainstream school and social inclusion. Among children on the autism spectrum, 36.2% attended mainstream school. These children showed significantly higher levels of social inclusion compared to non-attenders. Subgroup analysis based on the severity of autism symptoms revealed that the significant association remained in both the mild and moderate/severe subgroups, being more pronouced in the mild subgroup (*P*_for interaction_ < 0.001). Parents of children attending mainstream school reported greater social inclusion levels than those whose children did not; however, after adjusting for severity of autism symptoms and other factors, this association became non-significant. Our study demonstrates a positive association between attending mainstream school and social inclusion for children on the autism spectrum, highlighting the critical role of mainstream school education in fostering social opportunities and providing diverse learning resources. It also underscores the need for targeted support strategies for parents.

## Introduction

Autism is a lifelong neurodevelopmental condition characterized by early onset difficulties in social communication, repetitive and stereotyped behaviors, and narrow interests (American Psychiatric Association, 2013; World Health Organization, 2022). Globally, the prevalence of autism was 0.4% in 2019 (World Health Organization & United Nations Children’s Fund, 2023). Recent data from the Autism and Developmental Disabilities Monitoring (ADDM) Network program indicate that approximately one in 36 children has been diagnosed with autism in the U.S. (Centers for Disease Control and Prevention, 2023). In China, autism affects an estimated 0.7% of children under 14 years, totaling over two million, making it the most prevalent neurological and developmental disorder among children (Zhou et al., 2020).

Children on the autism spectrum often face difficulties in social and behavioral aspects, such as loneliness, reduced community involvement, stigma, discrimination, bullying, and exclusion (Ji et al., 2022; Lindsay & McPherson, 2012). These challenges can negatively impact their social skills, confidence, self-esteem, and overall life opportunities, potentially resulting in prolonged social isolation (Marsack & Perry, 2018). Strategies for social inclusion can help mitigate these consequences by improving social abilities, broadening social connections, boosting self-confidence, and combating discrimination (Edwards et al., 2022). Recognizing this, China’s State Council has emphasized the importance of supporting the integration of children on the autism spectrum into society (The State Council, 2018).

Autism not only impacts the social inclusion of children but also affects their parents, particularly mothers. Parents often make substantial personal sacrifices by leaving jobs and reducing social activities to provide care (Brisini & Solomon, 2020). In Chinese culture, the interdependent nature of family dynamics exacerbates these effects (Wang et al., 2023). Social inclusion is a complex and multidimensional process involving equal opportunities for social participation in both objective engagement and subjective experiences (Simplican et al., 2015). Numerous studies have shown that children on the autism spectrum and their families face notable challenges regarding social inclusion (Arias et al., 2018; Cameron et al., 2022; Holmes, 2024). However, there is a lack of quantitative research on social inclusion in China.

Simplican et al. (2015) proposed an ecological model of social inclusion that considers the various factors that impact it. In the case of children on the autism spectrum, schools are essential for their social inclusion as they offer crucial developmental environments and opportunities. However, public special education schools in China mainly cater to children with visual, hearing, and physical disabilities. As a result, many parents of children on the autism spectrum turn to private rehabilitation institutions for assistance (Ministry of Education of the People’s Republic of China, 2023; Tang & Liu, 2023). While special education can enhance communication and social skills, it may also restrict their access to mainstream education and real-world social interaction settings (Zheng et al., 2021). Inclusive education was first introduced globally in the 1994 Salamanca Statement (UNESCO, 1994), and later incorporated into China’s education policy framework (The State Council, 2014). It has become a pivotal strategy in addressing educational disparities for children with special needs. However, inclusive education is still in its early stages of development in China, commonly referred to as integrated education. It represents the main form of combining general education and special education, with ‘mainstreaming’ as the primary approach (The State Council, 2017).

Attending mainstream classes has been promoted as the primary approach for ensuring compulsory education for children on the autism spectrum (Li et al., 2021). Moreover, it has increasingly been adopted as a core rehabilitation goal by parents (Shenzhen ‘Dami & Xiaomi’ official account, 2018), reflecting a broader societal shift towards educational inclusivity and support for children with developmental differences. However, mainstream school non-attendance is common among these children in China (Totsika et al., 2020), compared to the higher rates of school attendance observed in developed countries (Department for Education of UK, 2024; Yao, 2020). A better understanding of the mainstream education status for children on the autism spectrum is needed to formulate better future policies.

Previous studies have mainly focused on the challenges faced by children on the autism spectrum in mainstream schools, such as academic struggles (Kong & Guo, 2020), anxiety (Zainal & Magiati, 2019), and emotional and behavioral issues (Hastings et al., 2021). However, there is limited literature on the benefits of attending mainstream school for children on the autism spectrum. Some research has highlighted improvements in home adjustment (Woodman et al., 2016) and socialization opportunities (Dahle, 2003). In promoting the social inclusion of children on the autism spectrum, inclusive education plays a crucial role (Beghin, 2021; Lynch & Irvine, 2009; Simón et al., 2023). Nevertheless, no studies have yet explored the relationship between attending mainstream school and the social inclusion of children on the autism spectrum and their parents in China.

This study aimed to investigate the association between attending mainstream school and social inclusion for children on the autism spectrum and their parents. The study considered the severity of autism symptoms and other relevant factors. We hypothesized that attending mainstream school would positively impact the social inclusion of children on the autism spectrum and their parents.

## Methods

### Study Procedure and Participants

This multicenter cross-sectional survey was conducted from July to August 2023 at 30 rehabilitation centers for children on the autism spectrum in Hunan Province, China. Parents who were primary caregivers of children with autism were asked to take part in the study. To be eligible, parents needed to have at least one child diagnosed with autism based on the DSM-5 criteria (American Psychiatric Association, 2013).

The survey was conducted in 14 prefecture-level divisions in Hunan Province by The Guardians of the Stars, a nonprofit project team consisting of 14 undergraduate nursing volunteers from the Nursing School of Hunan University of Chinese Medicine. Each participant was interviewed individually in a quiet and well-lit room during a single session. Parent participants provided written informed consent and answered demographic questions first. The autism diagnosis and severity questions were determined by the children’s psychiatrist based on DSM-5 criteria (American Psychiatric Association, 2013; Mehling & Tassé, 2016). The parents completed questionnaires with guidance from a research staff member, while another staff member cared for the child on the autism spectrum to allow parents to focus on the questionnaires. Each interview session lasted around 15–20 min.

Initially, 1,000 parent-child pairs were contacted, of which 888 agreed to participate and completed the survey (response rate: 88.8%). Twenty-eight pairs were excluded due to the children being either younger than 3 or older than 14. The final sample consisted of 860 parent-child pairs. The study protocol was approved by the Ethics Committee of Hunan Provincial Brain Hospital (No. 2023-K-001).

### Measures

#### Mainstream School Attendance

The parents were asked if their child attended a mainstream school (yes or no). A ‘no’ response means their child is solely receiving rehabilitation training at a rehabilitation center. A ‘yes’ response means their child is involved in integrated education. In China, integrated education for children on the autism spectrum includes half a day of rehabilitation training and half a day of compulsory education in a mainstream school. This is the primary model of integrated education for these children in China. Rehabilitation training is separate from mainstream education, with programs at rehabilitation centers tailored to address individual symptoms. This dual approach aims to bridge the gap between rehabilitation and educational settings in order to provide children with comprehensive support for social and academic success.

#### Sociodemographic Variables

The parents provided sociodemographic information including age, gender, employment status, marital status, educational level, place of residence, and average monthly family income. Information about their child was also gathered, such as age, gender, duration of autism, duration of rehabilitation training, whether the child was an only child, severity of autism symptoms (‘mild’, ‘moderate’, or ‘severe’), number of siblings, and presence of similar developmental diseases.

#### Social Inclusion of Children on the Autism Spectrum

The parents responded to the Chinese version of the social inclusion subscale (Ji et al., 2025) from the KidsLife-ASD Scale, developed by Gómez et al. (2018) to assess quality of life outcomes in children and adolescents with autism and intellectual disabilities aged 4–21 years. This scale consists of eight subscales: emotional well-being, material well-being, physical well-being, personal development, rights, self-determination, interpersonal relationships, and social inclusion (Schalock et al., 2002). The social inclusion subscale is commonly used to evaluate social inclusion in autistic populations, with demonstrated reliability and validity (Morán et al., 2019; Stone et al., 2019). The Chinese version of the social inclusion subscale includes 11 items rated on a 4-point frequency scale (‘never’, ‘sometimes’, ‘often’, and ‘always’) and has shown good reliability and validity (Ji et al., 2025) with a Cronbach’s α of 0.96 in this study.

#### Parents’ Social Inclusion

The Social Inclusion Scale (SIS), developed by Secker et al. (2009), and validated in a Chinese version by Ji et al. (2024), was utilized to measure social inclusion. It assesses social contact, social acceptance, participation in activities, and subjective feelings. The scale consists of 19 items rated on a 4-point Likert scale ranging from ‘not at all’ to ‘yes definitely’, reflecting relationships over the past month. The SIS is commonly used to evaluate social inclusion levels in students and individuals with intellectual disabilities (Baus et al., 2024; Miller et al., 2023; Wilson & Secker, 2015). Secker et al. (2009) emphasize the importance of validating the SIS within the general population. The Chinese version of the SIS has shown good reliability and validity (Ji et al., 2024), with a Cronbach’s α of 0.87 in the present study.

### Statistical Analysis

The normal distribution of continuous variables was confirmed through graphical and statistical analysis. Descriptive statistics included means and standard deviations (SDs) for continuous variables, and frequencies (%) for categorical variables. Differences in participants’ characteristics were compared based on mainstream school attendance (yes/no) using χ2 tests for categorical variables, and t-tests for continuous variables. The correlation between social inclusion of children and their parents was tested using the Pearson correlation coefficient.

General linear mixed models were utilized to explore the association between attending mainstream school and the social inclusion of children and their parents, with survey sites considered as random effects. Multicollinearity among the independent variables was assessed using a variance inflation factor test. A minimally adjusted model (Model 1) was created, including the child and parent’s age and gender. A comprehensive model (Model 2) incorporated all covariates, such as urban or rural residence, average monthly family income (< 3000 CNY, 3000–6000 CNY, 6000–9000 CNY, or > 9000 CNY), parent’s educational level (junior middle school or below, high school, or college or above), current employment status (yes or no), current marital status (yes or no), duration of autism (years), duration of rehabilitation training (months), autism symptom severity (mild, moderate, or severe), number of siblings in the household (0, 1, or ≥ 2), and presence of similar developmental disorders among siblings in the household (yes or no). The impact of autism symptom severity on social inclusion through mainstream school attendance was examined in this study by testing for heterogeneity in their associations. A multiplicative interaction term (mainstream school attendance × severity of autism symptoms) was used for testing the associations. Additionally, this interaction term was accounted for in Model 2. Subgroup analyses were performed based on severity of autism symptoms. Due to the limited number of children with severe autism symptoms (*n* = 106), the moderate and severe autism symptom subgroups were combined.

Statistical analyses were conducted using SAS software (version 9.4; SAS Institute, Cary, North Carolina, USA). Two-sided statistical tests were conducted, with *p* < 0.05 considered as statistically significant.

## Results

Overall, 36.2% of children on the autism spectrum attended mainstream school. The children had a mean age of 6.0 years (SD 2.1) with 19.8% being female. Parents had a mean age of 35.6 years (SD 5.1) with 88.1% being female. The mean social inclusion score for children was 23.9 (SD 6.0; interquartile range 20–27; range 11–44) and for parents was 54.3 (SD 8.4; interquartile range 50–58; range 28–76).

Table 1 displays the characteristics of parent-child pairs according to whether the child attended mainstream school. Children in mainstream school were older, had milder autism, lived in urban areas, and had higher family monthly income compared to those not enrolled in mainstream school. Parents of children in mainstream school tended to have higher educational levels.

**Table 1:** Characteristics of the study participants by mainstream school attendance

Characteristics	Overall (*n* = 860)	Attending a mainstream school		*p* value
		Yes (*n* = 311)	No (*n* = 549)	
Mean age of the child (SD), years	6.0 (2.1)	6.4 (2.2)	5.9 (2.0)	< 0.001
Gender of the child (female), %	19.8	19.3	20.0	0.79
Duration of autism (SD), years	3.7 (2.2)	3.8 (2.2)	3.7 (2.1)	0.88
Duration of rehabilitation (SD), months	31.2 (24.7)	31.1 (25.8)	31.2 (24.1)	0.96
Severity of autism symptoms, %				< 0.001
Mild	46.7	65.9	35.9	
Moderate	40.5	30.6	46.1	
Severe	12.8	3.5	18.0	
Mean age of the parent (SD), years	35.6 (5.1)	35.9 (5.1)	35.5 (5.2)	0.25
Gender of the parent (female), %	88.1	88.4	88.0	0.84
Place of residence (urban), %	61.1	69.5	56.3	< 0.001
Family monthly income, %				0.05
< 3000 CNY	17.6	13.5	19.9	
3000–6000 CNY	44.4	43.7	44.8	
6000–9000 CNY	20.3	23.8	18.4	
> 9000 CNY	17.7	19.0	16.9	
Parent’s educational level, %				0.002
Junior middle school or below	18.0	12.2	21.3	
High school	40.0	40.8	39.5	
College or above	42.0	47.0	39.2	
Currently employed (parent), %	29.5	32.8	27.7	0.11
Currently married (parent), %	96.2	96.8	95.8	0.48
Number of siblings in the household, %				0.054
0	35.5	40.2	32.8	
1	45.2	40.2	48.1	
≥ 2	19.3	19.6	19.1	
Siblings with similar developmental disorders (yes), %	3.1	2.3	3.6	0.26

Note: SD, standard deviation; CNY = Chinese yuan renminbi

**Fig. 1:**
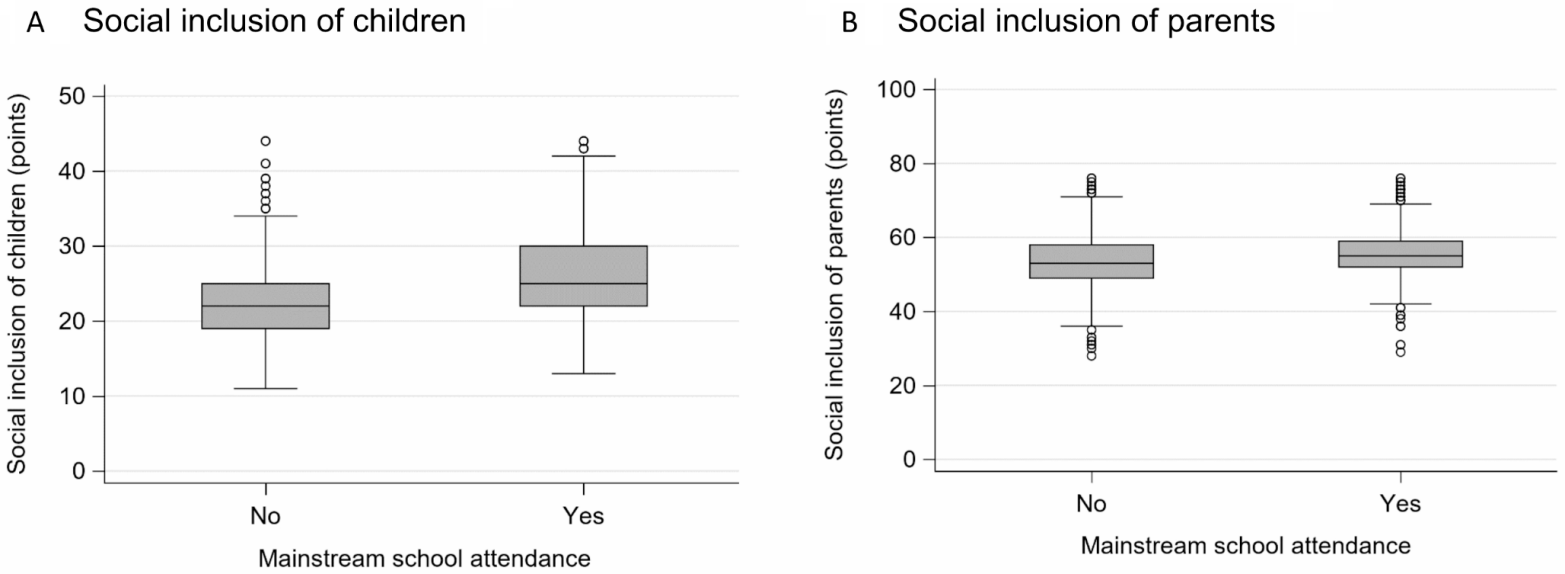
Box and whisker plot with the distribution of social inclusion according to mainstream school attendance in 860 children on the autism spectrum (**A**) and their parents (**B**) The boxes indicate the interquartile range (IQR) and the line in the center indicates the median score of social inclusion. Whiskers extend to 1.5 times the IQR to the most distant observation within that distance. Outlier observations are shown as circles

Figure 1 displays box plots showing the distribution of social inclusion scores among children on the autism spectrum and their parents based on attending mainstream school. Children attending mainstream school had significantly higher levels of social inclusion than their counterparts (mean [SD]: 26.2 [6.4] vs. 22.7 [5.4], *p* < 0.0001), as did their parents (55.5 [8.1] vs. 53.7 [8.6], *p* = 0.003). Similar trends were seen in two subscales of parents’ social inclusion: social contact (mean [SD]: 14.7 [3.0] vs. 14.1 [3.1], *p* = 0.006), participation in activities (mean [SD]: 22.2 [3.5] vs. 21.3 [3.7], *p* < 0.001), social acceptance (mean [SD]: 12.3 [2.2] vs. 12.1 [2.2], *p* = 0.07), and subjective feeling (mean [SD]: 12.6 [2.1] vs. 12.4 [2.2], *p* = 0.26). The Pearson correlation coefficient between children’ social inclusion and that of their parents was 0.48 (*p* < 0.001).

Table 2 displays the association between attending mainstream school and the social inclusion of children on the autism spectrum and their parents. Children in mainstream school showed significantly greater social inclusion, with an age- and gender- adjusted mean difference of 3.6 (95% confidence interval [CI]: 2.8–4.4; *p* < 0.001), compared to those in other settings. This association remained significant even after considering the severity of autism symptoms, the interaction between mainstream school attendance and symptom severity, and other factors (*p* = 0.008). Parents of children in mainstream school also exhibited higher levels of social inclusion, with an age- and gender- adjusted mean difference of 2.1 (95% CI: 0.9–3.2; *p* < 0.001). However, this difference were attenuated and became non-significant after adjusting for autism symptom severity and other factors (mean difference: 0.35 [95% CI: -1.5 to 2.2]), *p* = 0.71).

**Table 2:** Association between mainstream school attendance and social inclusion among children on the autism spectrum and their parents using linear mixed models

	Model 1*	*p* value	Model 2†	*p* value
	Least square means (95% CI)		Least square means (95% CI)	
**Children’s social inclusion**				
Not attending a mainstream school	22.6 (22.0–23.1)		22.8 (21.7–23.9)	
Attending a mainstream school	26.2 (25.5–26.9)		24.6 (23.1–26.1)	
Mean difference	3.6 (2.8–4.4)	< 0.001	1.7 (0.5–3.0)	0.008
**Parents’ social inclusion**				
Not attending a mainstream school	53.6 (52.8–54.2)		52.3 (50.2–54.4)	
Attending a mainstream school	55.6 (54.7–56.5)		52.0 (49.3–54.6)	
Mean difference	2.1 (0.9–3.2)	< 0.001	-0.3 (-1.5–2.2)	0.71

Note: CI: confidence interval

* Model 1 was adjusted for the age and gender of the children and parents

† Model 2 was additionally adjusted for place of residence (urban or rural), average monthly family income (< 3000 CNY, 3000–6000 CNY, 6000–9000 CNY, or > 9000 CNY), parent’s educational level (junior middle school or below, high school, or college or above), currently employed (yes or no), currently married (yes or no), duration of autism (years), duration of receiving rehabilitation training (months), severity of autism symptoms (mild, moderate, or severe), the interaction term of mainstream school attendance × severity of autism symptoms, number of siblings in the household (0, 1, or ≥ 2), and whether any siblings have similar developmental disorders in the household (yes or no). The survey site was modeled as a random effect

A significant interaction was observed between attending mainstream school and the severity of autism symptoms in relation to the social inclusion of children (*p* < 0.001). This interaction did not apply to the parents. Table 3 presents the findings of the subgroup analysis that investigated the association between mainstream school attendance and children’s social inclusion based on the severity of their autism symptoms. Mainstream school attendance was associated with greater social inclusion among children in both the mild and moderate/severe subgroups (both *p* values ≤ 0.01), with a more pronounced effect noted in the mild subgroup.

**Table 3:** Subgroup analysis of the association between mainstream school attendance and the social inclusion of children on the autism spectrum by disease severity using linear mixed models

	Model 1*	*p* value	Model 2†	*p* value	*p*_for interaction between mainstream school attendance and severity of autism symptoms_ ‡
	Least square means (95% CI)		Least square means (95% CI)		
**Mild Autism** (*n*** = 402)**					< 0.001
Not attending a mainstream school	23.9 (22.9–25.0)		24.4 (22.0–26.9)		
Attending a mainstream school	27.5 (26.5–28.6)		28.2 (25.6–30.7)		
Mean difference	3.6 (2.3–4.8)	< 0.001	3.7 (2.4–5.0)	< 0.001	
**Moderate or Severe Autism** (*n*** = 458)**					
Not attending a mainstream school	21.8 (21.1–22.4)		20.9 (19.1–22.6)		
Attending a mainstream school	23.3 (22.2–24.4)		22.2 (20.3–24.2)		
Mean difference	1.5 (0.4–2.6)	0.007	1.4 (0.3–2.4)	0.01	

Note: CI: confidence interval. The groups with moderate and severe autism were combined because the sample size of the severe group was small

* Model 1 was adjusted for the age and gender of the children and parents

† Model 2 was additionally adjusted for place of residence (urban or rural), average monthly family income (< 3000 CNY, 3000–6000 CNY, 6000–9000 CNY, or > 9000 CNY), parent’s educational level (junior middle school or below, high school, or college or above), currently employed (yes or no), currently married (yes or no), duration of autism (years), duration of receiving rehabilitation training (months), number of siblings in the household (0, 1, or ≥ 2), and whether any siblings have similar developmental disorders in the household (yes or no)

‡ P for interaction was tested with adjustments for all covariates in Model 2

## Discussion

In this cross-sectional study of Chinese children on the autism spectrum and their parents, we discovered that attending mainstream school was associated with increased social inclusion in children but not in parents. The results highlight the importance of attending mainstream school for children on the autism spectrum. This is one of the few studies examining the relationship between mainstream school attendance and social inclusion in children on the autism spectrum and their parents.

Our research revealed a positive association between attending mainstream school and social inclusion for children on the autism spectrum, irrespective of the severity of their symptoms, supporting a previous study (Dahle, 2003). Two other studies focusing on school students investigated the association between social inclusion and educational achievement/sense of belonging at school. A study conducted in Australia discovered that higher levels of social inclusion were associated with an increased likelihood of completing school (Renner et al., 2024). Another study in Finland found that social inclusion mediated the relationship between school belonging and depressive symptoms in students aged 13–18 (Haddadi Barzoki, 2024). Belongingness and social inclusion are fundamental psychological needs in social settings, promoting better opportunities for meaningful interactions and close relationships (Leemann et al., 2022). These studies show that social inclusion, educational attainment, and mental health are interrelated. While attending mainstream school alone does not guarantee social inclusion, it can help provide access to educational opportunities and a sense of belonging (Mannion, 2003). This feeling of belongingness can serve as a source of learning and support in stressful times (Bennett et al., 2018). Additionally, compared to private rehabilitation institutions, mainstream schools offer children on the autism spectrum more social opportunities and diverse learning resources (Lu et al., 2023). This study is one of only a few that focuses on social inclusion and school learning for children on the autism spectrum. Our findings stress the importance of encouraging attendance at mainstream schools and implementing targeted interventions to increase social inclusion for these children. Embracing inclusive practices can enhance the educational experience for all students by promoting diversity and understanding.

Having a child on the autism spectrum attend a mainstream school was initially associated with higher levels of parental social inclusion. However, this association was not significant after considering the severity of autism symptoms and other factors. This aligns with a previous study by Liu (2021), indicating that mainstream schools in China often lack resources to help children on the autism spectrum, especially those with severe symptoms. As a result, parents must get more involved in the classroom, limiting their social interactions. In countries where assistant or resource teachers specialize in supporting these children in mainstream schools, parents can feel a significant relief from the caregiving burden (Hamid et al., 2020; Wang, 2022). Nonetheless, the shortage of special education teachers in mainstream schools in China (Xu, 2023) may explain why parents’ social inclusion did not improve with their children’s attendance at these schools. Our findings emphasize the importance of implementing effective strategies to support parents and reduce their classroom involvement. This includes updating teacher training to better serve students with different learning needs and advocating for the role of educational assistants in mainstream classrooms (Carpenter et al., 2015).

One notable finding of this study was the significant interaction between attending mainstream school and the severity of autism symptoms in relation to children’s social inclusion levels. This indicates that mainstream schools may be more beneficial for children with mild autism compared to those with moderate to severe symptoms, who may need additional support. Currently, the Chinese government has not established standardized criteria for enrolling children on the autism spectrum in mainstream schools, which contributes to schools commonly rejecting these children in China (Xu, 2020). This highlights the importance of having scientifically based and reasonable admission criteria for mainstream schools to ensure that children with moderate to severe autism symptoms are not at a disadvantage (Hastings et al., 2021; Kong & Guo, 2020; Zainal & Magiati, 2019). It is crucial that private rehabilitation institutions tailor targeted training programs for children with moderate to severe autism symptoms to prepare them for mainstream school admission. Moreover, alternative rehabilitation programs should be developed to support students whose needs are not fully addressed by mainstream schools. It is essential to understand that attending mainstream school does not always ensure inclusivity. The quality of the educational environment plays a crucial role. Building strong partnerships between special and mainstream schools by collaborating and sharing expertise is important (Shaw, 2017).

### Limitations

This study has some important limitations. First, data were collected at a single point in time, which hinders our understanding of changes in social inclusion over time. A longitudinal study would offer a more comprehensive perspective. Second, while this was a multicenter study, all participants were recruited from rehabilitation facilities, potentially limiting the generalizability of the findings to all children on the autism spectrum, particularly those without access to rehabilitation training. Third, the sample may have insufficiently represented fathers, suggesting that future studies should aim to capture their perspectives. Fourth, this study solely focused on the relationship between mainstream school attendance and the social inclusion of children on the autism spectrum and their parents; future research should delve deeper into school characteristics. Additionally, the study excluded children on the autism spectrum younger than 3 years and older than 14 years, potentially missing out on unique experiences not captured in this research. Finally, it is crucial to note that attendance in mainstream school does not automatically ensure social inclusion. Further research is necessary to explore effective strategies to help autistic children and their families enhance social inclusion.

## Conclusion

Our study shows a positive association between attending mainstream school and social inclusion for children on the autism spectrum. It emphasizes the crucial role mainstream school education plays in providing social opportunities and diverse learning resources. Our results indicate that children with mild autism may benefit more from inclusion compared to those with moderate to severe symptoms, suggesting a need for standardized admission criteria and tailored rehabilitation programs in China. Initially, attending mainstream school was associated with increased parental social inclusion, but this association became non-significant when considering autism symptom severity and other factors. This emphasizes the importance of creating effective support strategies for parents and decreasing their involvement in the classroom.

## Abbreviations

ADDM: Autism and Developmental Disabilities Monitoring
CI: Confidence interval
SD: Standard deviations
